# Stress-Induced Ultrasonic Vocalization in Laboratory Rats and Mice: A Scoping Review

**DOI:** 10.3390/brainsci14111109

**Published:** 2024-10-31

**Authors:** Anumitha Venkatraman, Michelle Bretl, Se-in Kim, Leslie Christensen, Cynthia A. Kelm-Nelson, Michelle R. Ciucci, Susan L. Thibeault

**Affiliations:** 1 Division of Otolaryngology—Head & Neck Surgery, Department of Surgery, University of Wisconsin-Madison, Madison, WI 53705, USA; venkatraman@surgery.wisc.edu (A.V.); bretlm@surgery.wisc.edu (M.B.); kimse@surgery.wisc.edu (S.-i.K.); cakelm@wisc.edu (C.A.K.-N.); ciucci@surgery.wisc.edu (M.R.C.); 2 Ebling Library for the Health Sciences, University of Wisconsin-Madison, Madison, WI 53705, USA; leslie.christensen@wisc.edu

**Keywords:** stress, ultrasonic vocalization, stress paradigm, rat, mouse

## Abstract

**Introduction**: Ultrasonic vocalization (USV) can indicate affective states—including psychosocial stress—in mice and rats. However, stress-induced USV changes could be confounded by laboratory experimental variables such as the type of behavioral stress paradigm, the elicitation method, rodent strain, etc. We sought to provide a review of the current literature to delineate how psychosocial stress-altered rodent USVs may be affected by factors of age, sex, strain, species, elicitation paradigm, and stressor. **Methods**: We used PubMed, Scopus (Elsevier), PsycINFO (EBSCO), and the following Web of Science (Clarivate) databases: Biological Abstracts, CAB Abstracts, Science Citation Index-Expanded, and Emerging Sources Citation Index. The studies identified by our search strategy were independently screened by two authors with the following inclusion criteria: peer-reviewed, in English, reported original data, and described USV in response to stress in rats or mice. The data extracted included USV acoustic parameters (mean peak frequency and mean amplitude (loudness)), details of the stress and USV elicitation paradigms, rodent species, age, and sex variables. **Results**: The following screening of 5309 titles/abstracts and 687 full-text articles revealed 148 articles. Footshock (20%), cold exposure (14%), and maternal separation (23.5%) were the most commonly used stress paradigms (duration and type of stressor varied across studies), with the total number of USV calls being the most commonly reported acoustic outcome. In rats, 121 articles described stress-altered USVs, while 25 studies reported the same in mice, and two reported multiple rodent species (rats and mice, alongside other rodent species such as gerbils). With respect to stress-altered USV changes with age, mice and rats increase USV rates after birth, with a peak around 6 to 10 days, and decrease USVs until weanling age. Of the five studies that reported sex-related differences in stress-induced USVs, females had an increased number of calls and lower average peak frequency in response to stress when compared to males. Only two to four studies reported strain-related differences in stress-induced vocalizations in rats and mice, respectively. **Conclusions**: The data from this review lay the groundwork for better understanding rodent USVs in response to psychosocial stress with effects of elicitation paradigm, stressor, age, and sex.

## 1. Introduction

Vocalization is critical for social interactions across vertebrate species; however, little is known about how stress affects vocal behavior. Social rodents—including mice and rats—vocalize in ultrasonic ranges (above 20 kilohertz (kHz)) for communicative intent, commonly referred to as ultrasonic vocalization (USV) [1,2]. Three types of USVs have been documented and differ in temporal and frequency parameters [3] to indicate affective states (aversive calls (distress), positive affective calls, and pup calls in response to maternal separation) [4]. Specifically, adolescent and adult rats and mice emit lower frequency calls (~22 kHz) in response to aversive or anxiogenic stimuli [5,6,7,8] and higher frequency calls in response to mating or tickling (~50 kHz) [9]. Lower frequency aversive calls may be further characterized by their function, such as “alarm cries”, versus defensive cries in response to another rodent [10]. Due to developmental differences in the rodent pup larynx, pups emit higher frequency calls to indicate an aversive state when separated from their mother (~40–60 kHz) [11,12,13]. All USVs are produced by a glottal jet impinging on the thyroid inner wall during exhalation in both mice and rats [14]. Further, studies in rats (but not mice) have shown that USV is regulated by intrinsic laryngeal muscle activity via subglottal pressure [15,16]. The prior literature has also described similarities between the acoustic patterns of rodent ultrasonic vocalizations and human infant cries in response to aversive stimuli [17].

Mice and rat ultrasonic vocalizations share acoustic similarities in their spectrogram; the average fundamental frequency falls in the range of 20–95 kHz, the average call duration ranges from 30 to 60 ms, and the amplitude or intensity of calls can easily differentiate them from background noise; pause duration (if present) is less than 20 ms [18,19]. USV calls can also be classified based on complexity. Complex calls are multi-syllabic, with each individual syllable lasting 5–150 ms [18,19]. Complex and simple positive affective USVs in rats can further be classified into 14 subcategories based on frequency modulation (complex, upward and downward ramp, flat, short, split, step-up, step-down, trill, multi-step, trill, flat/trill combination, inverted U, composite, and trill with jumps) [18,19]. Similar categorizations of mice ultrasonic vocalization calls have resulted in the following subcategories; (upsweep, downsweep, half, full, and two cycles, harmonic, multiple jumps, jump down, and jump up) [20]. Negative affective USVs are thought to be “flat” and less complex [18,19]. In addition to the USV call subcategory, USV spectrograms can also be described by the number of calls, call rate (number of calls/min), mean peak frequency, call duration, average intensity or amplitude, and call bandwidth [20,21].

Psychosocial stress is defined as an unusual or intense level of stress that is induced due to an altered social situation [22]. Rodents are often used as models of stress, as inducing intense levels of stress in humans for the purpose of studying physiological mechanisms of stress is not always possible. Intense and repeated stress exposure increases the risk of mental health conditions, such as anxiety, depression, and post-traumatic stress disorder [23]. Ultrasonic vocalizations from laboratory rats and mice can be used to better understand the behavioral responses to medications, such as selective serotonin reuptake inhibitors, used to treat various mental health conditions [24]. Anxiety can co-occur with Parkinson’s disease, expressed in the USVs of the genetic rat model of early-onset PD (*Pink1*-/-) [25], which has been known to have vocal deficits in communication similar to humans [26]. Overall, the physiological and morphological similarities between a rodent (rat and mouse) larynx and a human larynx [15,16] make rodents an ideal model for relating behavioral responses to underlying physiological changes caused by stress exposure.

A number of rodent stress paradigms, including social defeat, cold exposure, novel social environment, and others, have been developed to understand the biological and physiological mechanisms of psychosocial stress. However, the effect of the type and duration of stressor/s has rarely been compared across studies [27], especially with regard to modulation vocalization properties. Increased duration of stress exposure, depending on the type of stressor, does lead to persistent USV changes in mice and rats, even following stressor cessation [13,20]. Certain types of stressors are more aversive (e.g., restraint stress) than others (e.g., novel environment), which results in different stress-induced USV outcomes. Moreover, there are different responses to psychosocial stress paradigms based on sex, age, or strain [14,15,16,28]. Female rats produce aversive USV calls of shorter duration when compared to male rats in response to laboratory stressors such as restraint stress [15,16]. Male pups produce a lower average peak frequency of aversive USVs compared to female pups when both sexes are separated from their mother [29]. With respect to age, rodent pups increase the number of calls [30,31,32,33] when compared to juvenile rodents in response to stress [34]. Meanwhile, mice pups, in the first few days of life, may produce no USVs in response to cold stress [35]. C57BL/6J pups produce a decreased number of aversive calls compared to other mouse strains, indicating potential strain-specific differences in stress-induced USVs [32,36]. Most importantly, the techniques or methods of eliciting USVs, such as a mating paradigm or social isolation, are specific to different affective states (positive and aversive states, respectively) [37,38], thus resulting in different USV outcomes.

The goal of this scoping review is to describe stress-induced rodent USV in different experimental stress paradigms and USV elicitation techniques while considering species, strain, age, and sex in laboratory rats and mice. The data from this report will lay the groundwork for carefully selecting and executing psychosocial stress paradigms in laboratory rats and mice for the study of mechanisms of the laryngeal-respiratory system in response to psychosocial stress.

## 2. Methods

This scoping review follows the Preferred Reporting Items for Systematic Reviews and Meta-Analyses Extension for Scoping Reviews (PRISMA-ScR) reporting guidelines [39].

The review team collaborated with a University of Wisconsin-Madison research librarian to develop and execute a comprehensive search of the literature. This search combined controlled vocabulary and title/abstract terms related to USV in rats and mice. Search strategies were also peer-reviewed by three UW-Madison Science and Veterinary Medicine librarians. The detailed search strategies are available in Supplemental Material S1. Searches were run on 12 September 2022, from database inception through the present in the following databases: PubMed, Scopus (Elsevier), PsycINFO (EBSCO), and the following Web of Science (Clarivate) databases: Biological Abstracts, CAB Abstracts, Science Citation Index-Expanded, and Emerging Sources Citation Index. No date, language, or publication type filters were applied to the results. The results were downloaded to citation management software (EndNote Version 20) and underwent manual deduplication via the librarian using the method described by Bramer et al. [40]. Unique records were uploaded to a screening platform (Covidence) for review by team members.

### 2.1. Screening Procedures

Two authors independently screened the title and abstracts of each record for eligibility using the criteria stated below. Identified articles that were deemed appropriate for inclusion were then subjected to a rigorous full-text review independently by two authors using the same eligibility criteria as the title and abstract screening process. Any conflicts during the screening process were resolved by consensus.

### 2.2. Eligibility Criteria for Study Inclusion

Peer-reviewed articles in English that described USV following a stress-inducing paradigm in rats and mice were included. We defined a stress-inducing paradigm as one in which controlled psychosocial stress was induced under laboratory conditions. Proof of psychosocial stress induction was provided by (1) prior citations that support the use of that paradigm for studying psychosocial stress and/or (2) quantifiable stress-induced behavioral changes (using Light/Dark Box, Elevated Plus Maze, Sucrose preference testing, freezing behavior, etc.) [11,41,42,43], and/or stress-induced physiological changes (i.e., increased in corticosterone levels indicating hypothalamic-pituitary-adrenal axis) [30,41,44]. Studies were excluded if they did not use rats or mice, the animals had a prior medical condition or pharmaceutical drug treatment that confounded the effects of psychosocial stress (epilepsy), or the animals experienced stress prenatally prior to USV collection. Articles were also excluded if USV or elicitation paradigms were not described or the effects of psychosocial stress on USV could not be delineated (i.e., lack of a within or between-subject control comparison).

### 2.3. Data Extraction and Synthesis of the Results

The following data were extracted from relevant articles following full-text screening: first author, year, title, details on frequency of reporting, the type and duration of each stress paradigm, age, sex, species and strain-related variables studied, elicitation paradigm of USV, and the corresponding stress-altered USV outcomes (including the effect of stress persistence on USV).

## 3. Results

**Figure 1:**
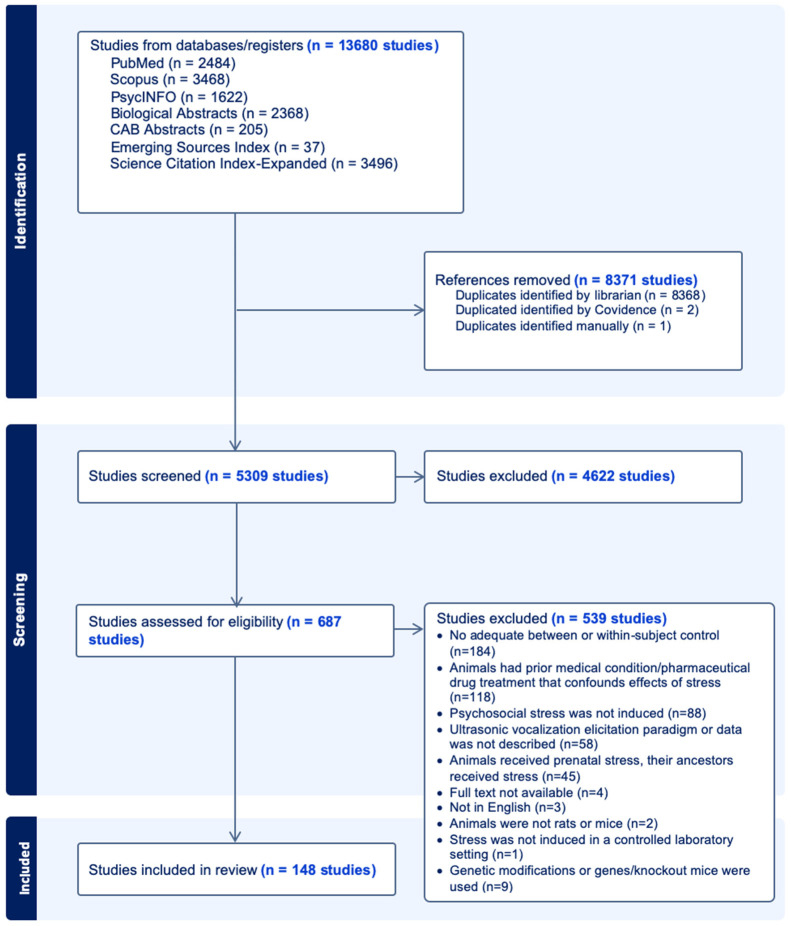
PRISMA Flow Diagram.

Of the 5309 unique records identified in the database search after deduplication, 4622 records were removed during title and abstract screening, 687 articles underwent full-text review, and 148 met the inclusion criteria and were included in this review (Figure 1).

### 3.1. Frequency of Reporting of Different Stress Paradigms

The 148 articles included in this review spanned the years 1971–2022. The number of studies increased with increasing decade (1971–1980: 6, 1981–1990: 9, 1991–2000: 29, 2001–2010: 48, 2010–2020: 57, 2020–Present: 8; Figure 2). Of these studies, 37 articles included physiological measures to confirm stress, 64 articles included behavioral measures of stress [45,46], and the remaining 47 articles reported citations of previous studies that validated the stress-inducing paradigm.

The three most common stress-inducing paradigms were maternal separation (23.5%, most commonly studied across all time periods), footshock (20%, most commonly studied from 2001–2020), and cold exposure (14%, most commonly studied from 1971–1980, 1991–2000, and 2011–2020). The least common stress-inducing paradigms were blue light exposure, intermittent cold swim stress test [47], and food/water deprivation and chasing (0.6%). The remaining stress-inducing paradigms were reported with a frequency between 2% and 8% (predator odor, acoustic stress, chronic variable stress, novel social and housing environment, social defeat, and whisker clipping/ear notching). While studies containing other stress-inducing paradigms may exist in the literature, they did not fit our inclusion criteria.

### 3.2. Effect of Age, Sex, and Species-Related Variables on Stress-Induced USVs

We identified particular patterns of stress-altered USV in mice of different strains and ages.

(a) Species and strain-related changes in USVs:

Of the 148 articles included in the full-text review, 121 of the articles utilized rats, 25 articles utilized mice, and two articles utilized some combination of rodent species; for example, one study compared five rodent species, including ICR mice, Wistar Imamichi rats, Mongolian gerbil, Syrian hamster, and a vole strain [38]. One discernible difference for species is that the stress paradigms that used mice were focused on social stress, including isolation (24%), maternal/littermate separation (68%), or restraint stress (8%) to measure stress-induced USV. In contrast, studies that used rats employed aversive stress environments to measure stress-altered USV, such as social defeat (9%). Specifically, 34 rat studies used footshock (with or without fear conditioning) as the elicitation technique compared to the single study with mice.

The most commonly used strains of mice include C57BL/6 (13 studies) and CBA/CaJ (4 studies), with the remaining mouse studies reporting a variety of strains. Two studies compared different strains of mice: BTBR T+ tf/J mouse pups emitted significantly more isolation-induced USV than C57BL/6J mouse pups under clean and soiled bedding conditions (Table 1) [36]. Cold exposure stress resulted in a high rate of USVs (calls/min) in C3H/HeJ and compared to C57BL/6J pups, with BALB/cJ pups vocalizing at a rate in between C3H/H3J and C56BL/6J pups [32].

The most commonly used strains of rats include Sprague-Dawley rats (53 studies) [48,49,50], Wistar rats (40 studies), and Long-Evans rats (18 studies), with other strains utilized in the remaining reported studies (10 studies; Holtzman, Lister Hooded, etc.). However, only four of these studies compared stress-induced USVs in more than one strain of rats (Table 1). Of these four studies, Lister Hooded rats were noted to have the longest call duration of USVs from a single session of footshock, followed by Long-Evans rats and Wistar rats, with Sprague Dawley rats having the shortest USV call duration [24]. When exposed to social defeat, Sprague-Dawley rats reportedly produced a significantly greater number of USVs than non-stressed controls, while Wistar rats showed no significant differences in the number of USVs produced between rats experiencing social conflict and those who did not (non-stressed controls) [9]. However, in response to isolation in a novel cage, Wistar rats produced more calls than Long-Evans rats [36]. Lastly, Wistar-Kyoto rats emitted the same average number of USVs (in response to stressors, isolation, and a maze), while Wistar rats showed a decreased number of USVs in the maze context compared to isolation [51]. Four additional studies used rats but did not specify the strain(s) [12,52,53,54].

(b) Age-related changes in USVs:

Most studies have reported an increased number of USVs from birth for a certain number of days, followed by a decrease to nearly zero by weaning age, if not earlier (post-natal, 14–21 days of age) [13,30,38,55,56,57,58,59]. Many studies indicated that stress-induced USV rates (number of calls/min) peaked between 6 and 10 days of age [30,31,32,33]. One study reported an earlier spike in the number of USVs, around 4–6 days old, with an earlier decrease in the number of USVs, between 6–8 days old, then remaining low through 14 days old [60]. Only one study commented on changes in USV call rate (calls/min) in the weanling and late adolescent stages, reporting decreased latency to call (or time taken to begin calling after the stimulus was presented) from 22 to 54 days old and a peak in vocalization duration at weanling age (28 days old), which declined sharply to late adolescence (37 days old) [34]. Between 1–7 days of age, the number of USVs increased, with no USVs at 21 days of age [33,60,61,62].

(c) Sex-related changes in stress-altered USVs:

Five studies that used both male and female rodents did not report sex-related differences in stress-altered USV. Six studies found that there were no differences/effects of sex as it relates to USVs [12,56,61,63,64]. Five studies reported significant sex-related differences in stress-induced USVs, but the measurement of sex-related changes in USVs differs across studies. One study noted sex differences as it relates to USV response to an acoustic startle, where females did not produce any calls in response to an acoustic startle [65]. Average peak frequency aversive vocalizations is a USV outcome that demonstrated sex differences, where males produced higher peak frequency than females [66,67]. The duration of calls has also been reported as an outcome, though the acoustic parameters are inconsistent. Depending on the stress context, males seemingly had greater USV durations in response to fear conditioning [37], while females who experienced maternal deprivation had greater duration in response to isolation [13]. Most frequently, the number of USV calls has been utilized as a USV outcome that has demonstrated sex-related changes; three studies reported that stress results in fewer USV calls from males compared to females [13,37]. Rather than the number of total calls, one study reported that females emitted fewer “fragmented” calls than males, regardless of stress [57]. Finally, two studies could not comment on clear sex-related differences in USVs due to confounding variables such as age and stressors [57,68]. Although we did not include prenatal stressors within the inclusion criteria for our scoping review, juvenile female mice may have strain-specific USV differences in response to prenatal stressors [69].

### 3.3. Effect of Type and Duration of Stress Paradigm on USVs

Stress paradigms reported in three or more studies were investigated for patterns in the type and duration of stressors and their effect on stress-induced USVs.

(a) Restraint Stress: Four studies involved restraint stress. The type of restraint used for inducing stress varied, but most involved a customized modification of a conical-shaped tube [70,71]. Overall, the average number of positive affective USVs was reduced by restraint stress on each of the 7 days that rats were restrained when compared to baseline (~17–30 calls/15 s post-stress compared to 35–45 calls/15 s pre-stress) [70,71]. C56BL/7 mice had increased amplitude, bandwidth [72], and number of calls [35] with restraint.
(b) Predator/Predator Odor: Eleven studies used predator/predator odor [66,73,74,75,76,77]. Six studies report an increase in the duration of time spent vocalizing in response to either predator exposure (cat) or predator odor (ferret bedding) for 20–25 min total (~20–60% time vocalizing with exposure compared to ~10% without exposure) [75,76]. This response habituates over time [75]. Neonates have a decreased number of USVs from 200–250 calls to positive affective calls in response to predator odor [77]. In one study, the number of pups per litter affected USV response to a 5 min exposure to predator odor (three-pup litters decreased call amplitude, and two-pup litters increased call amplitude) [66].
(c) Chronic Variable Stress: Four studies used chronic variables for stress in rats, which is a combination of different stressors. The stressors differed in duration (4–6 weeks) and type (wet bedding, loud noise, light exposure, water deprivation, cage tilting, novel housing environment, restraint, cage tilt, forced swim, and elevated platform) [45,78,79,80]. In all included studies, the number of 50 kHz calls decreased, and the number of 22 kHz calls increased in response to stress [45,78,79,80].
(d) Cold Exposure: A total of 21 studies used cold exposure; this stress paradigm was more common in pups [81,82]. Of note, C56BL/7 mice that are 3 days to 14 weeks of age, as well as Wistar pups, did not emit alarm-related USVs following cold stress at 2–10 °C for 7–12 days, but Sprague Dawley and albino rat pups increase USV production in response to cold (e.g., 87–205 calls/2 min) [33,35,81,83,84,85,86,87,88]. Sprague Dawley pups experienced a habituation effect (i.e., USVs reduce the number of calls by 21 days when stress is administered from 3 days) [89],
(e) Social Defeat: Twelve studies used social defeat as a stressor. The increased duration of the paradigm increased the number of aversive calls and decreased the number of positive affective calls [9,44,90,91,92,93,94].
(f) Novel Social or Housing Environment: Fourteen studies involved novel social and housing environments. Light source (dim), increased familiarity with social conspecifics, and prior history or current isolation can negatively affect the number of USVs (increase aversive calls and decrease positive affective calls) [66,81,95,96,97,98,99,100].
(g) Maternal Separation: Thirty-four studies employed maternal separation [12,13,31,42,53,57,58,61,65,88,101,102,103,104,105,106,107,108,109,110,111,112,113,114,115,116,117,118,119] in a temperature-controlled incubator to reduce attrition [12,13,31,42,53,57,58,61,65,102,103,104,105,106,107,108,109]. In some cases, USVs are measured during the separation period (2–13 days) or when an anesthetized dam or littermates are reintroduced back into the cage [12,13,31,42,53,57,58,61,65,102,103,104,105,106,107,108,109]. Overall, pups increase their vocalization rate (calls/min) at aversive calls (24–250 calls in 1–10 min intervals) [12,13,31,42,53,57,58,61,65,102,103,104,105,106,107,108,109]. Three report a decrease in the number of aversive calls [56]. Dams also increased the number of positive affective calls in response to maternal separation [65,105,120]. Pups reduce their USV response to stress once reunited with their mother [120]. The results on call duration and call repertoire varied across studies, strains, and breeding lineage [56,120].
(h) Footshock: Twenty-nine studies paired electric footshock with acoustic tone, an odor, or a treat (e.g., grape) for fear conditioning in rodents [22,24,43,121,122,123,124,125,126,127,128,129,130,131,132,133,134,135,136,137]. Varied doses of current and duration of footshock are reported in the literature (0.04–3 mA of current, administered for 1–2 s, at various intervals for up to 70 trials or 40 min). All studies report a decrease in the number and duration of aversive calls [22,24,43,121,122,123,124,125,126,127,128,129,130,131,132,133,134,135,136].

### 3.4. Effect of Elicitation Paradigm on Stress-Induced USVs

Portfos et al. (2007) [4] have described both positive and negative affective calls that not only differ in temporal and frequency characteristics but utilize different elicitation paradigms. In contrast, positive affective calls may be elicited via a mating paradigm or social contact, and aversive calls may be elicited via the stressor itself or an additional acoustic startle stimulus. Of the 148 total articles, 105 studies reported only aversive USVs [138,139,140,141,142,143,144], 25 studies reported only positive affect-related USVs [145], and the remaining 18 studies reported both aversive and positive affect-related USVs [146]. We will discuss the elicitation paradigms associated with each of these separately.

### 3.5. Aversive Calls

(a) Acoustic startle/Air puff [147]: Four studies utilized auditory/acoustic stress in order to elicit USVs. Two of these studies utilized acoustic startle elicitation in conjunction with a maternal separation stress paradigm [65,105]. Two studies utilized startle-inducing acoustic stimuli and found that there were continuous USVs throughout testing [148,149], with a potential habituation effect. Ten additional studies utilized a fear conditioning paradigm with an acoustic/auditory cue, air puff, either with USV playback, noise, or tone, with inconsistent effects due to confounders [121,124,127,128,133,134,150,151,152,153].
(b) Isolation: Twenty-nine studies utilized isolation as the primary elicitation paradigm [154], which differed from the stress paradigm. Of these, five studies utilized brief isolation as both the stressor and elicitation procedure [12,62,155,156,157,158,159].
(c) Stressor: Eighty articles utilized the stressor as the elicitation paradigm. In response to the stressor, 75% (24 of 32 studies) reported an increase in lower frequency calls in infant rodents. The effect on the 22 kHz USVs appeared to be dependent on the stressor and could be confounded by many other variables, including age and sex, as discussed above.

### 3.6. Positive Affective Calls

(a) Social contact: Seven studies utilized some variation of social contact as the elicitation paradigm for positive affective USVs. The types of social contact included a mating paradigm (where USVs were measured post-ejaculation and during male-female interaction) [22,160], same-sex exposure (juvenile rough and tumble behavior) [5,6,7,8], or tickling [37,161].
(b) Isolation: Only five studies utilized isolation as the elicitation procedure for 40–50 kHz USVs. The magnitude and change in isolation-emitted 40–50 kHz vocalization reported in response to stress varied across studies, with some rodent studies reporting an increased number of higher frequency USVs (~40–50 kHz for rat pups and 55 kHz in mice) [38,53].
(c) Stressor: Twenty-nine studies utilized the stressor as the elicitation paradigm. The effect on positive affective USVs is variable depending on the stressor. Sixty-six percent of these studies (10/15) reported an increase in the number of higher-frequency calls in infant rodents.

### 3.7. Effect of Stress Persistence on USV

For the purposes of this review, we define stress persistence as the continued observation of stress-altered USVs when measured after stress cessation, also known as fear memory 1 or retention 2. A wide variety of stress paradigms—with different durations, species, and ages—have been used to study stress persistence [162]. Overall, longer stress paradigms resulted in longer-lasting effects of stress persistence (with stress persistence measured anywhere from 1 h to multiple days following stress cessation) [3,4]. One exception is when the stress paradigm involves footshock. In this case, fear duration or increased electric shock amplitude did not necessarily correspond with longer stress persistence [13,14,15,16,17,18,19,20]. Out of 22 studies that reported on the effect of stress persistence on USV, nine studies noted that USV generally returns to baseline within 7 days of stress cessation (regardless of the duration or type of the initial stressor). Few studies report strain-specific age and sex-related effects on stress persistence in rats and mice; however, no consensus exists.

## 4. Discussion

The goal of this scoping review was to survey the literature on rodent USVs in response to psychosocial stress paradigms. A variety of psychosocial stress paradigms are described in our 148 reported studies (from 1971 to 2022); however, maternal separation, cold exposure, and footshock were the most common across multiple decades. Overall, there were 4.84 times more studies using rats to study stress-altered USVs when compared to studies using mice, with few studies (two–five studies) reporting on each strain and sex-related differences in stressed USVs. In response to stress, mice and rat pups generally increase the number of USVs after birth, peaking around 6 to 10 days, and decrease the number of USVs until 21 days of age, when pups are generally weaned. A large variation in the type and duration of stress paradigms and USV elicitation paradigms has been reported across studies.

Experimental protocols and procedures for maternal separation, cold exposure, and footshock stress paradigms have been well-established and well-described, facilitating easy replicability across studies [86,102,107,126]. Other stress paradigms developed in the last decade (2011–2020) are more difficult to replicate; protocols vary across reported studies, such as chronic variable stress or chasing stress, which are reported in 0.6% of the studies in this review [163,164]. One reason that some experimental stress paradigms are more common than others could be that the Institutional Animal Care and Use Committee (IACUC) has stringent regulations for the types of stress-related procedures that can be conducted in rodents [165], which could dissuade researchers from using stress paradigms involving painful and aggressive interactions between mice and rats. The IACUC regulations are based on the United States Department of Agriculture Animal Welfare Act, which was amended in 1985 to classify experimental rodent stress paradigms as highly painful [165], which could have further reduced the number of studies incorporating certain stressful paradigms after 1985. Stress paradigms that involve painful and aggressive interactions between mice and rats include novel social housing and social defeat (reported in studies from 1991–2020, constituting 2–8% of reported studies in our review) [44,166,167].

Much of the reported literature focuses on stress-altered USVs in rats rather than mice (4.84 times more reported studies in rats). Unlike rats, mice do not produce ultrasonic vocalizations in response to certain types of stressors, such as those involving aggressive encounters or tail pinching [4]. From a mechanistic perspective, studies have shown that rat USVs are influenced by intrinsic laryngeal muscle activity via subglottal pressure [15,16], but the same has not been studied in mice. As such, rats produce more complex USVs than mice, which involve several acoustic components within a single call [14]. Rats are generally preferred for studying behavioral responses, as they are more social animals compared to mice [168]. Only a few studies have compared stress-altered USV calls across different strains of rats and mice [7,32,36,80,169]. There are also confounding factors related to how investigators classify USV calls or analyze individual acoustic parameters within calls and the type of stress paradigm used during experiments. Overall, more studies and comparisons across studies are necessary to better understand physiological differences among strains and species as they relate to USV production.

The USV frequency range lowers in adult rats (~22 kHz) compared to infants (40–60 kHz), similar to what is seen in humans [170]. However, while 14 studies report the effects of age on stress-induced USVs, these studies used mice and rat pups below 21 days of age (before weaning from the mother) [13,30,38,55,56,57,58,59]. Only one study compared rats with ages ranging from early to late adolescence (22–54 days of age) [34]. Positive stimuli were more commonly used in infant rats and mice to elicit USVs (five studies used social play or tickling as the elicitation paradigm) [5,6,7,8,37,161]. This was less common in adult rats and mice (only two studies used the mating paradigm to elicit USV) [22,160]. The choice of elicitation paradigm and age groups in these studies may be to prevent stress-induced attrition in young pups and older adult mice and rats.

Although sex hormones can have an effect on rodent intrinsic musculature and vocal fold mucosa [171], only five studies reported sex-related differences in stressed USVs (out of 13 studies using both male and female rodents). In these five studies delineating sex-related differences, females have an increased number of calls and decreased average peak frequency when compared to males in response to stress. Sexual dimorphism in rodent laryngeal physiology has mainly been studied in ovariectomized or pregnant rats and mice [171], which were not the female rodent population in our reported studies. 

The majority of stress paradigm studies reported in our scoping review show an increase in negative and positive affective USVs in adult rodents as a result of stress, regardless of elicitation paradigms. In this section, we will discuss how the type of stress paradigm may affect stress-induced USVs. Restraint stress in Sprague Dawley rats results in a fear-associated elevation in respiratory rate [172], which can affect or reduce the production of positive affective USVs [173]. As with restraint stress, predator odor/predator exposure can also increase respiratory rate in rats [76], which is associated with an increase in aversive USVs. Aversive calls are thought to be longer in duration but less complex than positive affective calls. Social defeat paradigms involve physical confrontation and are more severe than restraint stress and predator odor/exposure but are also associated with a reduced number of positive affective calls (as well as increased aversive calls) compared to undefeated control animals. In the case of social defeat, stress-induced respiratory changes can persist for up to 30 days following stress cessation [167]. Given that a large majority of USVs return to baseline within 7 days of stress cessation, more research is required to relate both physiological and functional acoustic parameters in stress persistence across different stress paradigms. One exception is the footshock stress paradigm; studies report that expiration duration is correlated with call duration, with the respiratory rate increasing rapidly for a short period of time with the onset of a novel stimulus [76]. Following conditioning, however, these animals may exhibit a lower respiratory rate during vocalizations prior to footshock conditioning [76]. This differing respiratory pattern in response to footshock could contribute to the differing pattern of stress persistence/effects, wherein increased shock amplitude or duration of footshock does not affect the duration of stress persistence. With maternal separation, studies report that the paraventricular nucleus exerts control over stress-altered respiratory physiology, thereby exhibiting long-term effects on maternally separated pups as they progress to adulthood [174]. Overall, respiratory system regulation plays a role in the majority of stressed USV responses across different paradigms.

Chronic variable stress uses combined aspects of other reported stress paradigms; however, the duration and type of stressor in these paradigms make it difficult to determine the role of each stressor (or combination) to the resulting stress-induced USVs [45,78,79,80]. Similarly, novel social and housing environments are studied in a variety of different contexts. On the other hand, cold exposure has a relatively consistent duration and type of stressor but results in different USVs in different strains of mice and rats [35,81,83,84,85,86,87,88]. With respect to underlying physiology, cold stress can increase oxygen consumption (and prolonged expiration rather than inspiration) [85]. Authors have termed the maneuver to increase oxygen consumption and cardiac output in response to cold stress as “abdominal compression reaction” or “laryngeal braking” [175], which leads to an increase in aversive USVs in these animals (particularly in Sprague Dawley rats) [85,86].

USVs are broadly thought to be representative of rodent affective states. As one would expect from stress paradigms (anxiogenic stimuli), aversive calls were more commonly measured across the reported studies [35,81,83,84,85,86,87,88]. In general, aversive calls are generally considered less complex calls compared to positive affective calls [30]. For both negative and positive affective USV, the number of USV calls [66,95,96,97] was the most commonly reported outcome, followed by the call duration and latency of USV calls from the initiation of the stressor [74,75]. When taken together, various psychosocial stress paradigms have been successfully used to elicit USV in mice and rats. However, the varied duration and type of stress paradigm, as well as the elicitation paradigm reported across studies, makes comparisons of USVs difficult. To truly identify the effect of these variables, controlled prospective studies are required.

## 5. Conclusions

In this review, we have summarized 148 studies that utilize a wide variety of psychosocial stress paradigms to elicit USV in mice and rats. Of the different stress paradigms, maternal separation, cold exposure, and footshock were reported as the most common across multiple decades (during the years 1971–2022). There were 4.84 times more studies that used rats to study stress-altered USV when compared to studies using mice, with few studies reporting strain-related differences in these rodents (two to four studies with mice and rats, respectively). With respect to age, mice and rats increase the number of stressed USVs for the first 6 to 10 days of life and then decrease the number of stressed USVs until 21 days of age (when pups generally need to be weaned from their mother). Five studies showed that females have an increased number of calls and decreased average peak frequency in response to stress when compared to males. The type and duration of the stressor and USV elicitation paradigm vary across studies. This review lays the groundwork for standardizing psychosocial stress paradigms and USV call outcome (call rate and mean peak frequency) analysis such that additional comparisons can be made across studies in the future.

## Figures and Tables

**Figure 2:**
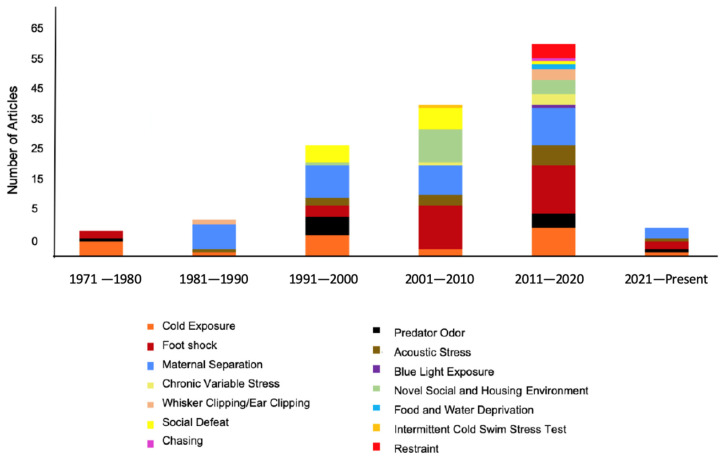
Graphical Representation Depicting Number of Articles Incorporating Various Stress Paradigms Across Multiple Decades.

**Table 1:** Stress Induced USV Outcomes Reported for Articles Comparing Strains of Mice and Rats.

Author, Year	Age	Strain/s	Stress Paradigm	USV Elicitation Technique	Stress-Induced USV Outcomes	
**Mice**						
Nitschke, 1972 [33]	Infant	C57BL/6J, BALB/cJ, C3H/Hej mice	Cold exposure	Cold exposure	C56BL/6J	Reduced USV call rate over time, with no calls after 6 days of stress
					BALB/cJ	Reduced USV call rate over time, with no calls 12 days of stress
					C3H/Hej	Maintained relatively high USV call rate over time, even after 12 days of stress
Woehr, 2015 [32]	Infant	BTBR T+tf/J, C57BL/6J	Isolation in a novel context (soiled and clean bedding)	Isolation	BTBR T+tf/J	Higher # of USVs in soiled and clean bedding compared to C57BL/6J Reduced # of USVs with soiled bedding compared to clean
					C57BL/6J	No change with bedding type
**Rats**						
Kassai, 2018 [34]	Adult	Sprague Dawley, Wister, Long Evans Lister-Hooded	Footshock (single session)	Footshock	Sprague Dawley	Lowest USV call duration
					Wister	Higher USV call duration compared to Sprague Dawley
					Long Evans	Same as Wister rats, higher USV call duration than Sprague Dawley
					Lister Hooded	Highest USV call duration
Walker, 2009 [8]	Adult	Sprague Dawley, Wister	Social Defeat	Social Defeat	Sprague Dawley	Increased # of USVs compared to Wistar rats and non- stressed controls
					Wister	No change in # of USVs compared to non-stressed controls
Woehr, 2008 [35]	Adult	Long Evans, Wistar	Isolation (novel cage)	Isolation	Wistar	Decreased # of USVs in novel cage compared to home cage Increased # of USVs compared to Long Evans
					Long Evans	Decreased # of USVs in novel cage compared to home cage
Rao, 2015 [36]	Adult	Wistar Kyoto, Wistar	Isolation and Elevated Plus Maze	Isolation and Elevated Plus Maze	Wistar Kyoto	Produced longer call duration in isolation compared to maze No change in # of USV between isolation and maze
					Wistar	Produced longer call duration in isolation compared to maze Decreased # of USV in maze compared to isolation

## Supplementary Materials

The following supporting information can be downloaded at: https://www.mdpi.com/article/10.3390/brainsci14111109/s1. Supplemental File S1: Search Strategy Information.
