# Supplementary material for: Stress-Induced Ultrasonic Vocalization in Laboratory Rats and Mice: A Scoping Review
Source: Brain Sci. 2024 Oct 31;14(11):1109. doi: 10.3390/brainsci14111109 (PMC11591760; doi:10.3390/brainsci14111109)
Supplement: Supplementary file 1 [file brainsci-14-01109-s001.zip › brainsci-3262708-supplementary.pdf]

Supplemental File S1: Search Strategy Information

Conducted by Librarian: Leslie A. Christensen, MA-LIS
leslie.christensen@wisc.edu | ORCID ID: 0000-0002-4040-5593
Ebling Library for the Health Sciences, University of Wisconsin - Madison

Database	Search Run Date	Results
**PubMed**	**09/12/2022**	2,484
**Scopus (Elsevier)**	**09/12/2022**	3,468
**Biological Abstracts (WOS)**	**09/12/2022**	2,368
**CAB Abstracts (WOS)**	**09/12/2022**	205
**Emerging Sources Citation Index (WOS)**	**09/12/2022**	37
**Science Citation Index-Expanded (WOS)**	**09/12/2022**	3,496
**PsycINFO (EBSCO)**	**09/12/2022**	1,622
**Total**		13,680
**Total after librarian deduplication**		5,312
**Total after Covidence deduplication**		5,310

PubMed Search

("Vocalization, Animal"[mesh] OR (vocal*[tiab] OR communicat*[tiab] OR call[tiab] OR calls[tiab] OR calling*[tiab])) AND (“Acoustics”[mesh] OR "Ultrasonic Waves"[mesh] OR "Ultrasonics"[mesh] OR (USV[tiab] OR ultrasonic*[tiab] OR ultra-sonic*[tiab] OR ultrasound*[tiab] OR ultra-sound*[tiab] OR acoustic*[tiab] OR 22khz*[tiab] OR 50khz*[tiab] OR ((22[tiab] OR 50[tiab]) AND (khz[tiab] OR khzs[tiab] OR kilohertz[tiab] OR kilo-hertz[tiab])) OR ((low[tiab] OR lower[tiab]) AND (frequenc*[tiab])))) AND ("Murinae"[mesh] OR (murinae*[tiab] OR apodemus*[tiab] OR mastomy*[tiab] OR praomy*[tiab] OR acomy*[tiab] OR bandicota*[tiab] OR rat[tiab] OR rats[tiab] OR rattus[tiab] OR mus[tiab] OR musculus*[tiab] OR mouse[tiab] OR mice[tiab]))

Scopus
(TITLE-ABS-KEY(vocal* OR communicat* OR call OR calls OR calling*)) AND (TITLE-ABS-KEY(USV OR ultrasonic* OR ultra-sonic* OR ultrasound* OR ultra-sound* OR acoustic* OR 22khz* OR 50khz* OR ((22 OR 50) W/3 (khz OR khzs OR kilohertz OR kilo-hertz)) OR ((low OR lower) W/3 (frequenc*)))) AND (TITLE-ABS-KEY(murinae* OR apodemus* OR mastomy* OR praomy* OR acomy* OR bandicota* OR rat OR rats OR rattus OR mus OR musculus* OR mouse OR mice))

Web of Science (Clarivate): Biological Abstracts
(TS=(vocal* OR communicat* OR call OR calls OR calling*)) AND (TS=(USV OR ultrasonic* OR ultra-sonic* OR ultrasound* OR ultra-sound* OR acoustic* OR 22khz* OR 50khz* OR ((22 OR 50) NEAR/3 (khz OR khzs OR kilohertz OR kilo-hertz)) OR ((low OR lower) NEAR/3 (frequenc*)))) AND (TS=(murinae* OR apodemus* OR mastomy* OR praomy* OR acomy* OR bandicota* OR rat OR rats OR rattus OR mus OR musculus* OR mouse OR mice))

Web of Science (Clarivate): CABI: CAB Abstracts
(TS=(vocal* OR communicat* OR call OR calls OR calling*)) AND (TS=(USV OR ultrasonic* OR ultra-sonic* OR ultrasound* OR ultra-sound* OR acoustic* OR 22khz* OR 50khz* OR ((22 OR 50) NEAR/3 (khz OR khzs OR kilohertz OR kilo-hertz)) OR ((low OR lower) NEAR/3 (frequenc*)))) AND (TS=(murinae* OR apodemus* OR mastomy* OR praomy* OR acomy* OR bandicota* OR rat OR rats OR rattus OR mus OR musculus* OR mouse OR mice))

Web of Science (Clarivate): Emerging Sources Citation Index
(TS=(vocal* OR communicat* OR call OR calls OR calling*)) AND (TS=(USV OR ultrasonic* OR ultra-sonic* OR ultrasound* OR ultra-sound* OR acoustic* OR 22khz* OR 50khz* OR ((22 OR 50) NEAR/3 (khz OR khzs OR kilohertz OR kilo-hertz)) OR ((low OR lower) NEAR/3 (frequenc*)))) AND (TS=(murinae* OR apodemus* OR mastomy* OR praomy* OR acomy* OR bandicota* OR rat OR rats OR rattus OR mus OR musculus* OR mouse OR mice))

Web of Science (Clarivate): Science Citation Index-Expanded
3,496 Results

(TS=(vocal* OR communicat* OR call OR calls OR calling*)) AND (TS=(USV OR ultrasonic* OR ultra-sonic* OR ultrasound* OR ultra-sound* OR acoustic* OR 22khz* OR 50khz* OR ((22 OR 50) NEAR/3 (khz OR khzs OR kilohertz OR kilo-hertz)) OR ((low OR lower) NEAR/3 (frequenc*)))) AND (TS=(murinae* OR apodemus* OR mastomy* OR praomy* OR acomy* OR bandicota* OR rat OR rats OR rattus OR mus OR musculus* OR mouse OR mice))

PsycINFO (EBSCO)
1,622 Results

((TI(vocal* OR communicat* OR call OR calls OR calling*) OR AB(vocal* OR communicat* OR call OR calls OR calling*) OR SU(vocal* OR communicat* OR call OR calls OR calling*)) AND (TI(USV OR ultrasonic* OR ultra-sonic* OR ultrasound* OUR ultra-sound* OR acoustic* OR 22khz* OR 50khz* OR ((22 OR 50) N3 (khz OR khzs OR kilohertz OR kilo-hertz)) OR ((low OR lower) N3 (frequenc*))) OR AB(USV OR ultrasonic* OR ultra-sonic* OR ultrasound* OUR ultra-sound* OR acoustic* OR 22khz* OR 50khz* OR ((22 OR 50) N3 (khz OR khzs OR kilohertz OR kilo-hertz)) OR ((low OR lower) N3 (frequenc*))) OR SU(USV OR ultrasonic* OR ultra-sonic* OR ultrasound* OUR ultra-sound* OR acoustic* OR 22khz* OR 50khz* OR ((22 OR 50) N3 (khz OR khzs OR kilohertz OR kilo-hertz)) OR ((low OR lower) N3 (frequenc*)))) AND (TI(murinae* OR apodemus* OR mastomy* OR praomy* OR acomy* OR bandicota* OR rat OR rats OR rattus OR mus OR musculus* OR mouse OR mice) OR AB(murinae* OR apodemus* OR mastomy* OR praomy* OR acomy* OR bandicota* OR rat OR rats OR rattus OR mus OR musculus* OR mouse OR mice) OR SU(murinae* OR apodemus* OR mastomy* OR praomy* OR acomy* OR bandicota* OR rat OR rats OR rattus OR mus OR musculus* OR mouse OR mice)))

Exemplar Articles (All retrieved)

(33808441[uid] OR 34439589[uid] OR 18417174[uid])
